# Awareness and knowledge of antimicrobial resistance and factors associated with knowledge among adults in Dessie City, Northeast Ethiopia: Community-based cross-sectional study

**DOI:** 10.1371/journal.pone.0279342

**Published:** 2022-12-30

**Authors:** Wudneh Simegn, Getachew Moges

**Affiliations:** 1 Department of Social and Administrative Pharmacy, School of Pharmacy, College of Medicine and Health Sciences, University of Gondar, Gondar, Ethiopia; 2 Department of Pharmacy, College of Medicine and Health Sciences, Wollo University, Dessie, Ethiopia; Xiamen University - Malaysia Campus: Xiamen University - Malaysia, MALAYSIA

## Abstract

**Background:**

Antimicrobial resistance is an important global health challenge. The current study aimed to assess the level of awareness and knowledge of antimicrobial resistance and factors associated with knowledge among adults in Dessie City, Ethiopia.

**Methods:**

A community-based cross-sectional study was conducted among 407 adults in Dessie City from June to July 2021. A systematic random sampling technique was used to select respondents, and Google Form was used to collect data online. The data was analyzed by SPSS Version 26. The associated factors of knowledge of antimicrobial resistance were identified by using bivariate and multivariable logistic regression. Independent variables with a P-value <0.2 were selected as candidate variables for multivariable logistic regression. Those variables with a P-value <0.05 were declared statistically significant factors.

**Result:**

Out of the required sample sizes, four hundred and seven participants were enrolled, giving a response rate of 99.3%. One hundred and fifty-two (37.3%) respondents were females. Nearly one-third of the respondents (28.3%) have taken antibiotics in the last 6 months. In this study, 73.7% of study participants were aware of the existence of germs; 58.2% were aware of the existence of antibiotic resistance to bacteria; 47.7% were aware of the existence of drug resistance; 39.8% were aware of the existence of antimicrobial resistance; and 36.6% were aware of the existence of antibiotic resistance. Sixty-four (15.7%) respondents were not aware of any of the above terms. Sixty (14.7%) of the respondents were not aware of any risk factor for antimicrobial resistance. About 63 (15.5%) of the respondents did not know the consequences of antimicrobial resistance. Two hundred and thirty-eight (58.5%) respondents had good knowledge of antimicrobial resistance. In this study, being male (AOR = 1.99; 95% CI: 1.23,3.20), college and above educational level (AOR = 3.50; 95% CI: 1.08,11.39), grade 11–12 educational level (AOR = 3.73; 95% CI: 1.20,11.61), getting advice from health professionals about how to take antibiotics (AOR = 1.84; 95% CI:1.07,3.17), using health professionals as a source of information on antibiotics (AOR = 2.51; 95% CI: 1.48,4.25), and taking antibiotics without prescription (AOR = 1.86; 95% CI: 1.04,3.30) were significantly associated with good knowledge of antimicrobial resistance.

**Conclusion:**

The study identified low awareness and knowledge of antimicrobial resistance among adults. Being male, higher educational level, getting advice from health professionals about how to take antibiotics, using health professionals as a source of information on antibiotics, and taking antibiotics without a prescription were significantly associated with good knowledge of antimicrobial resistance. Educational campaigns would be highly desirable for the public to improve their awareness and knowledge of antimicrobial resistance.

## Introduction

As it is defined by the World Health Organization (WHO), appropriate use of antimicrobials is the cost-effective use of antimicrobials that maximizes clinical therapeutic effect while minimizing both drug-related toxicity and the development of resistance [1,2]. Antimicrobial resistance is becoming the main global health challenge [3–6]. Globally, drug resistance causes an estimated 700,000 deaths each year [7,8]. It is predominantly high in low and middle income countries and is partly related to high levels of antimicrobial use [7,9–14]. Besides, the environment is also increasingly affected by the global spread of clinically relevant antimicrobial resistant organisms [4,15], which impacts treatment outcomes [16,17]. The other major factors contributing to the emergency and spread of AMR are misuse and overuse of antimicrobial agents [12,18], high load of infectious diseases, a deprived infection-control policy, poor-quality medicines, inadequate knowledge of AMR, misdiagnosis, and lack of laboratories for antibiotic susceptibility tests [7,19–21].

Several educational campaigns have been implemented worldwide to reduce AMR with different levels of effectiveness [11,19,22,23]. The WHO is organizing a global movement to raise awareness of antibiotic resistance and encourage best practices among the public, health, and agricultural professionals to avoid the further emergence and spread of antibiotic resistance by implementing antimicrobial stewardship [9,18,24]. Due to the common prevalence of infectious diseases in Ethiopia [24,25], there is an irrational use of antibiotics by the public, patients, and healthcare providers [21,26]. Despite this, no national antimicrobial control system or policy has been implemented to reduce AMR [27].

Several studies have been conducted to assess awareness and knowledge of AMR among the public. A cross-sectional survey in the district of Sialkot, Pakistan reported that more than half of the community participants (55.6%) had poor knowledge of AMR [28]. A cross-sectional study done at Felege Hiwot Hospital, Ethiopia among patients showed that 42.3% had poor knowledge about AMR [29]. A recent study in Kemissie Town, Northeast Ethiopia, reported that 41.6% of the community had awareness of AMR [24].

Rational use of antimicrobials is the main strategy to prevent AMR [30]. To achieve this, measuring the level of awareness and knowledge of AMR among the public will play a great role. This will help to identify the gaps for taking measures to promote appropriate use of antimicrobials and prevent the further development of antimicrobial resistance. It will also be important to plan the implementation of educational campaigns by health sector administrators. As there were no previous local studies that investigated the awareness and knowledge of AMR among adults, the authors assessed the level of awareness and knowledge of AMR and factors associated with knowledge among adults in Dessie City, Ethiopia.

## Methods

### Study area, design, and period

A community-based cross-sectional study was conducted in Dessie City, Northeast Ethiopia. Dessie is a multi-ethnic City located in the South Wollo zone of Amhara Regional State, 401 km away from Addis Ababa, the capital City of Ethiopia. The projection plan commission reported that there would be 48,144 people in 2021. There were 20 urban and 8 rural Kebeles (smallest units of administration in Ethiopia), which were administered in the City. The City had five hospitals (one public referral, one public general, and three private hospitals), eight health centers, and twenty-seven private clinics. In the hospitals, there were AMR stewardship committees and an infection prevention committee. The committees in each hospital provided in-service training to the health professionals and an educational campaign to patients in the morning session about AMR, but there was no well-structured educational program. There is no educational campaign given outside of hospitals to the general public. The study was conducted from June 30 to July 30, 2021.

### Population

All residents living in Dessie City were used as the source population. The study population consisted of residents who were present at the time of data collection. Residents who were health professionals, those who were severely ill, those who were unable to speak, and those who had lived less than six months were excluded.

### Sample size calculation and sampling technique

**Fig 1:**
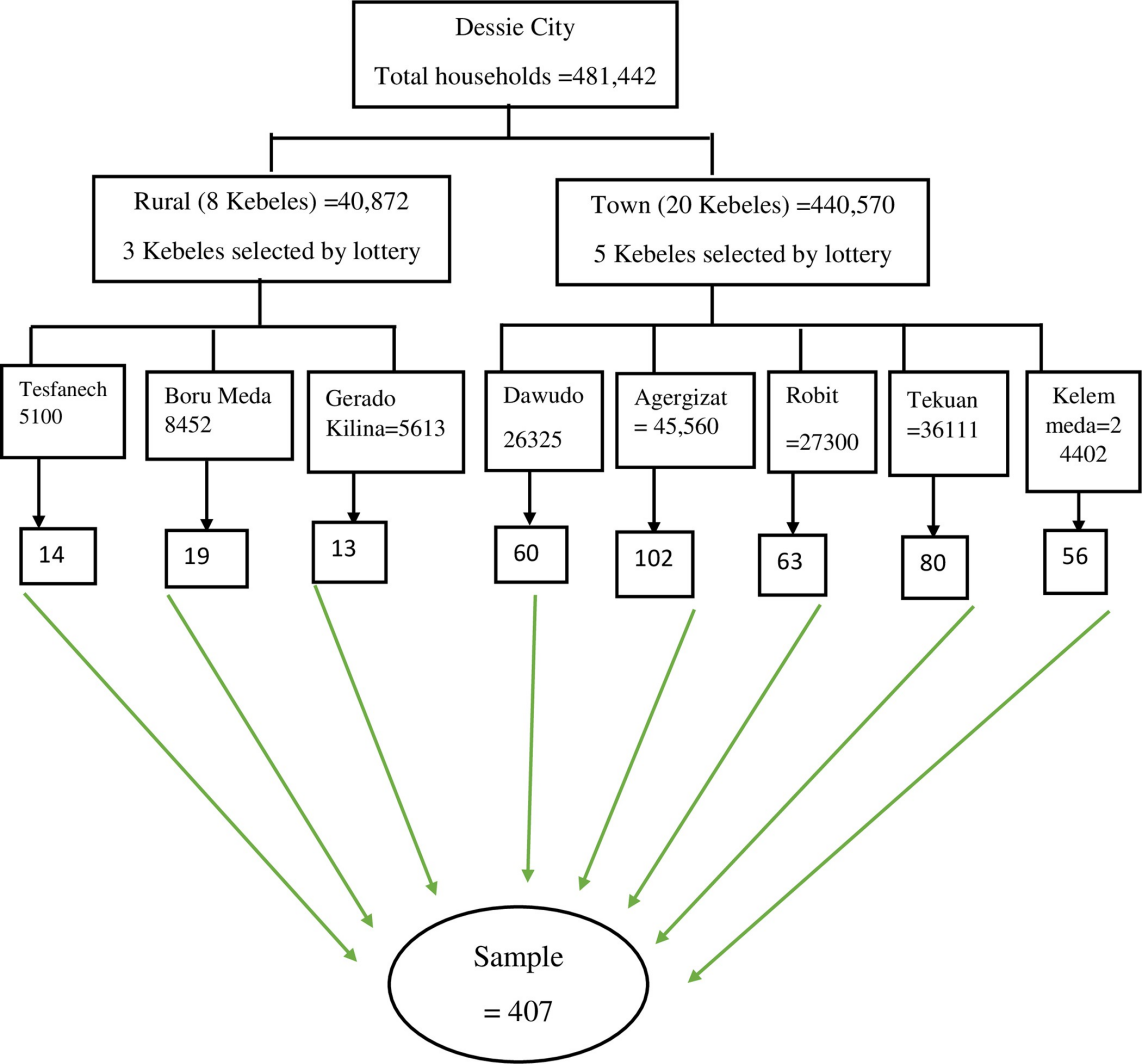
Schematic presentation of the sampling procedure for selecting study participants, Dessie City, north east Ethiopia, 2021 (n = 407).

The sample size was calculated using a single population proportion formula with the assumptions of prevalence of AMR awareness (41.6%) in the previous study [24], a 95% confidence interval and a margin of error (d) of 5%. After adding a non-response rate of 10%, the final sample size for this study was 410. We randomly selected 5 Kebeles (Dawudo, Ager Gizat, Robit, Tekuam, and Kelemmeda) from the total of 20 urban Kebeles in the City, and 3 Kebeles (Gerado Tesfanech, Boru Meda, and Gerado Kelina) from the total of 8 Kebeles in the rural parts. A stratified sampling technique was used to allocate the study participants proportionately to the urban and rural settings. The current number of households was taken from the City administration. The list of the households with their respective addresses was taken at each Kebele administrative office. From each stratum, samples were taken proportionally to their size by taking the number of households as the sampling frame. Finally, we used 361 study participants from the urban parts of the City and 46 study participants from the rural parts of the City. The households from each Kebele of an urban stratum and from each Kebele of a rural stratum were selected using a systematic random sampling technique. The sampling interval for each Kebele was determined by dividing the total number of households in each Kebele into its proportionally allocated sample size. Then, every K^th^ value of households was interviewed by randomly selecting the first household. The flow chart of sampling technique is included below (Fig 1).

### Data collection instrument and data collection procedure

The data was collected using a semi-structured interview-based questionnaire, which was adapted from the WHO level of antimicrobial resistance public awareness survey and another study [18,24]. The questionnaire included four parts. Part 1 included socio-demographic characteristics such as sex, age, educational level, occupation, and monthly income. Part 2 included items related to antibiotics use. Part 3 included questions used to assess awareness of antimicrobial resistance, including the risk factors and consequences of AMR. Part 4 included eight true/false questions to assess knowledge of AMR. As the data was collected during the peak COVID-19 outbreak, we used an electronic online Google form for data collection to minimize the spread of the pandemic. Two pharmacists were recruited to collect the data. They interviewed the eligible study participants and filled in the electronic online Google form using a smart phone. The data collectors contacted the head of the household for an interview. If the head of the household was not present at the time of data collection, any member of the household who was 18 years of age or older was recruited for the study. If any member of a household was not available during data collection, the next household was selected.

### Data quality control

A pretest was done among 20 residents outside of the study area to check the clarity of the questions, and we amended the few terms in the socio-demographic parts. The data collectors were trained about the purpose of the study and ethical issues in the process of data gathering. The principal investigator was actively involved in the data collection process. The questionnaire was prepared in English and translated to the local language (Amharic) and then back-translated into the English language by fluent speakers of the two languages to check the consistency.

### Variables of the study

Socio-demographic characteristics such as gender, age, residence, religion, marital status, educational level, occupation, and average monthly income were included. Information related to antibiotic use, such as the last time taking antibiotics, the sources of antibiotics, whether respondents got advice while taking antibiotics, the source of information on antibiotics, whether they suffered from different microbial infections, practice of self-medication with antibiotics, awareness, and knowledge of AMR were included.

### Measuring techniques

The measuring techniques for awareness of AMR describe the frequency and percentage score of each question. The measuring technique for knowledge of AMR was based on the World Health Organization (WHO) level of measurement of antimicrobial resistance among communities [18]. Respondents were provided with true or false answers to the eight questions, which were used to grade the respondents’ knowledge. A score of 1 was assigned for the correct responses and 0 for the incorrect ones. Then, the percentage score was determined. Study participants who scored less than 75% were considered to have poor AMR knowledge, while those who scored 75% and above were considered to have good AMR knowledge. This cut-off point was based on the national survey of public knowledge of AMR conducted in Nigeria [31].

### Terminologies

#### Antimicrobial resistance

Antimicrobial resistance occurs when bacteria, viruses, fungi and parasites change over time and no longer respond to medicines making infections harder to treat and increasing the risk of disease spread, severe illness and death [32].

#### Antibiotic resistance

Antibiotic resistance occurs when germs such as bacteria develop the ability to resist drugs that are designed to kill them [33].

#### Germs

Refers to the microscopic bacteria, viruses, fungi, and protozoa that can cause disease [34].

#### Drug resistance

The ability of disease-causing germs (e.g. bacteria or viruses) to continue multiplying despite the presence of drugs that usually kill them [32].

#### Antibiotic-resistant bacteria

The bacteria that are not killed by antibiotics and can continue to multiply presents a substantial threat to the control of infectious diseases [35].

### Statistical analysis

The data was analyzed using SPSS Version 26. The mean with standard deviation (SD) and frequency with percent were computed to describe the results of the study. Logistic regression was used to explain the association between knowledge of AMR and the independent variables. Independent variables with a p-value < 0.2 were selected as candidate variables for multivariable logistic regression. Variables with a p-value < 0.05 and 95% CI were treated as a significant factors for knowledge of AMR.

### Ethical approval and consent to participate

Ethical clearance was obtained from Wollo University, College of Medicine and Health Sciences, department of pharmacy Ethical review board with reference number: WU Phar/266/2013. A letter of cooperation was written to the Dessie City Administration office. Permission letters were obtained from each Kebele administration office. Written consent was obtained from each study participant. Confidentiality of the information collected was maintained by omitting any personal identifier from the data collection tool. This study was conducted according to the declaration of Helsinki.

## Results

### Socio-demographic characteristics of the study participants

In the current study, 407 study participants were enrolled with a response rate of 99.5% of which 152 (37.3%) were females. The mean age with standard deviation of the participants was 35.63±8.63 years and ranged from 20–68 years. Thirty (7.4%) participants were unable to read and write, while most of the participants attended grades 11–12 (30.2%). Majority (38.8%) of the study participants were merchants, followed by employees (24.3%) and housewives (16.2%) (Table 1).

**Table 1:** Socio-demographic characteristics of the study participants among adults in Dessie City, Northeast Ethiopia, 2021 (n = 407).

Variables	Categories	Frequency n%
Gender	Male	255(62.7)
	Female	152(37.3)
Age category	20–29	98(24.1)
	30–33	95(23.3)
	34–40	116(28.5)
	> 40	98(24.1)
Residence	Rural	46(11.3)
	Urban	361(88.7)
Religion	Orthodox	162(39.8)
	Muslim	175(43.0)
	Protestant	52(12.8)
	Catholic	18(4.4)
Marital status	Married	306(75.2)
	Single	62(15.2)
	Divorced	28(6.9)
	Widowed	11(2.7)
Educational level	Unable to read and write	30(7.4)
	Able to read and write	82(20.1)
	Grade 8–10	69(17.0)
	Grade 11–12	123(30.2)
	College and above	103(25.3)
Occupation	Employee	99(24.3)
	Merchant	158(38.8)
	Farmer	13(3.2)
	House wife	66(16.2)
	Student	22(5.4)
	No work	49(12.0)
Average monthly income (ETH-Birr)	0–2500	103(25.3)
	2501–4000	108(26.5)
	4001–6000	103(25.3)
	>6000	93(22.9)

### Reported antibiotics use by the study participants

Almost one-third (28.3%) of the respondents took antibiotics in the last 6 months. The majority of respondents got antibiotics from retail outlet pharmacies (66.3%). More than half (60.2%) of the respondents reported that they were advised by health professionals regarding antibiotics. The major sources of information about antibiotics were healthcare professionals (64.9%), followed by friends and family (46.2%), and previous experiences (38.3%). The majority (71.5%) of the respondents reported that they stop taking antibiotics when they have taken all the antibiotics as directed, while more than one-third (36.6%) reported that they discontinued their antibiotics when they felt better (Table 2).

**Table 2:** Antibiotic use and related information among the adults of Dessie City, Northeast Ethiopia, 2021 (n = 407).

Variables	Categories	Frequency n%
The last time to take antibiotics	In the last month	66(16.2)
	In the last 6 months	115(28.3)
	In the last year	114(28.0)
	More than a year ago	94(23.1)
	Cannot remember	18(4.4)
The sources of antibiotics (More than one answer is possible)	Hospital/healthcare by prescription	269(66.1)
	Retail outlet pharmacy	270(66.3)
	From a friend or family member	117(28.7)
	By sharing with others	61(15.0)
Getting advice from a doctor, nurse or pharmacist on how to take antibiotics	Yes	245(60.2)
	No	162(39.8)
The source of information on antibiotics (More than one answer is possible)	Healthcare professional	264(64.9)
	Mass media	78(19.2)
	Friends/ family	188(46.2)
	From previous experience	156(38.3)
Ever suffered from different microbial infection during lifetime	Yes	224(55.0)
	No	183(45.0)
Ever used antibiotics without prescription (self-medication with antibiotics)	Yes	225(55.3)
	No	182(44.7)
When do you think you should stop taking antibiotics once you have begun treatment? (More than one answer is possible)	Do not know	30(7.4)
	When I feel better	149(36.6)
	When I have taken all the antibiotics as directed.	291(71.5)
	When I encountered side effects	108(26.5)
	When forgetting	21(5.2)

### Awareness and knowledge of antimicrobial resistance

Of the total respondents included in this study, 73.7% of the respondents were aware of the existence of germs; 58.2% of the respondents were aware of the existence of antibiotic-resistant bacteria; 47.7% of the respondents were aware of the existence of drug resistance; 39.8% of the respondents were aware of the existence of antimicrobial resistance; and 36.6% of the respondents were aware of the existence of antibiotic resistance, while only 15.7% of the respondents were not aware of any of the existence of the above terms. Most of the respondents (70.8%) were aware that sharing antibiotics with others was a risk factor for AMR. Other risk factors mentioned by the respondents were failure to complete the course of therapy (61.4%), taking antibiotics without a prescription (54.3%), over or under use of antibiotics (43.7%) and taking antibiotics without considering the dose and time gap (40.5%). Sixty (14.7%) of the respondents were not aware of any of the risk factors for AMR. No cure for the diseases (77.1%), need for expensive drugs (63.9%), increased intensity and duration of the diseases (45.9%) and decreased antibiotic activity (45.2%) were the consequences of AMR reported by respondents. About 15.5% of the respondents never knew of any consequences related to AMR. In the current study, the mean knowledge score of all participants was 5.40 (95% CI: 5.28, 5.51). Two hundred and thirty-eight (58.5%) respondents had good knowledge of AMR (Table 3).

**Table 3:** Awareness and knowledge of AMR among the adults of Dessie City, Northeast Ethiopia, 2021 (n = 407).

Variables	Categories	Frequency	Percent (%)
When do you think, you should stop taking antibiotics once you have begun treatment? (More than one answer is possible)	Don not know	30	7.4
	When I feel better	149	36.6
	When I have taken all the antibiotics as directed.	291	71.5
	When I encountered side effects	108	26.5
	When forgetting	21	5.2
Do you heard/encountered the following terms? (More than one answer is possible)	Antibiotic resistance	149	36.6
	Drug resistance	194	47.7
	Antibiotic-resistant bacteria	237	58.2
	Germs	299	73.5
	Antimicrobial resistance	162	39.8
	None of the above	64	15.7
What do you think are risk factors of antimicrobial resistance? (More than one answer is possible)	Over or under use of antibiotic	178	43.7
	Failure to complete the course of therapy	250	61.4
	Sharing antibiotics with others	288	70.8
	Taking antibiotics without prescription	221	54.3
	Taking antibiotic without considering the dose and time gap	165	40.5
	I do not know	60	14.7
What do you think are the consequences of the antimicrobial resistance? (More than one answer is possible)	Decrease antibiotic activity	184	45.2
	Need for expensive drug	260	63.9
	Not cured from the diseases	314	77.1
	Increase intensity and duration of the diseases	187	45.9
	I do not know	63	15.5
knowledge of AMR	Good knowledge	238	58.5
	Poor knowledge	169	41.5

### Association of different factors with knowledge of antimicrobial resistance

Gender, age, residence, educational level, the last time to take antibiotics, using health facilities as a source of antibiotics, relying upon health professionals to get advice about how to take antibiotics, using health professionals as a source of information for antibiotics, relying upon previous experience for source of information for antibiotics, taking antibiotics without prescription, and suffering from different microbial infections were candidate variables for multivariable logistic regression (p-value<0.2).

In the final model, male study participants had 1.99-folds (AOR = 1.99; 95% CI: 1.23,3.20) better knowledge of AMR than females. Study participants with grade 11–12 educational level had 3.73 odds (AOR = 3.73; 95% CI: 1.20,11.61) and those with college and above educational level had 3.50 times (AOR = 3.50; 95% CI: 1.08,11.39) better knowledge of AMR than study participants who were unable to read and write. Study participants who were relying upon health professionals to get advice about how to take antibiotics had 1.84 times (AOR = 1.84; 95% CI: 1.07,3.17) better knowledge of AMR than those study participants who did not get advice from health professionals. Study participants who ever used health professionals as a source of information on antibiotics had 2.51 times (AOR = 2.51; 95% CI: 1.48,4.25) and those who used antibiotics without a prescription had 1.86 (AOR = 1.86; 95% CI: 1.04,3.30) better knowledge of AMR than their counterparts (Table 4).

**Table 4:** Association factors of knowledge towards AMR among the adults of Dessie City, Northeast Ethiopia, 2021 (n = 407).

Variables	Category	Knowledge of AMR		AOR (95% CI)
		Good %	Poor%	
Gender	Male	166(65.1)	89(34.9)	1.99(1.23,3.20)*
	Female	72(47.4)	80(52.6)	1
Age categories	20–29	63(64.3)	35(35.7)	0.95(0.46,1.99)
	30–33	59(62.1)	36(37.9)	1.02(0.50,2.05)
	34–40	69(59.5)	47(40.7)	1.26(0.66,2.43)
	> 40	47(48.0)	51(52.0)	1
Educational level	Unable to read and write	6(20.0)	24(80.0)	1
	Able to read and write	42(51.2)	40(48.8)	2.45(0.83,7.26)
	Grade 8–10	36(52.2)	33(47.8)	2.52(0.79,8.01)
	Grade 11–12	83(67.5)	40(32.5)	3.73(1.20,11.61)*
	College and above	71(68.9)	32(31.1)	3.50(1.08,11.39)*
Residence	Rural	21(45.7)	25(54.3)	1
	Urban	217(60.1)	144(39.9)	1.43(0.70,2.92)
When did you last take antibiotics	In the last month	43(65.2)	23(34.8)	1.01(0.28,3.61)
	In the last 6 month	78(67.8)	37(32.2)	1.66(0.50,5.51)
	In the last year	62(54.4)	52(45.6)	1.24(0.37,4.11)
	Before a year a go	48(51.1)	46(48.9)	1.04(0.32,3.43)
	Cannot remember	7(38.9)	11(61.1)	1
Getting antibiotics from health facility	Yes	172(63.9)	97(36.1)	1.46(0.89,2.42)
	No	66(47.8)	72(52.2)	1
Getting advice from health professionals about how to take antibiotics	Yes	171(69.8)	74(30.2)	1.84(1.07,3.17)*
	No	67(41.4)	95(58.6)	1
Using health professionals as source of information on antibiotics	Yes	187(70.8)	77(29.2)	2.51(1.48,4.25)*
	No	51(35.7)	92(64.3)	1
Using your previous experience as a source of information on antibiotics	Yes	103(66.0)	53(34.0)	1.10(0.66,1.81)
	No	135(53.8)	116(46.2)	1
Taking antibiotics without prescription	Yes	158(70.2)	67(29.8)	1.86(1.04,3.30)*
	No	80(44.0)	102(56.0)	
Suffering from different microbial infections during your life time	Yes	148(66.1)	76(33.9)	1.25(0.70,2.22)
	No	90(49.2)	93(50.8)	1

* p- Value < 0.05.

## Discussion

The current study assessed awareness and knowledge of AMR and factors associated with knowledge among adults in Dessie City, northeast Ethiopia. Nearly three-fourths of the respondents (73.5%) were aware of the term "germ". More than half of the study participants (58.2%) were aware of antibiotic resistant bacteria. Under half of the study participants were aware of antimicrobial resistance (39.8%) and antibiotic resistance (36.6%). The finding indicated a lower level of AMR awareness among the adults in Dessie City. The root causes of the low level of AMR awareness might be due to lack of information about the risk factors and the consequences of AMR, lack of engagement with the wider healthcare workforce, socio-economic factors and lack of an AMR educational campaign for the public [36–38]. Healthcare administrators should have an integrated approach by prioritizing AMR awareness creation and declaring the individual’s accountability could be the possible mechanism to bring changes in the awareness of AMR. To address the problems, increasing public education by using a nationwide television, radio, and social media campaign might be expected. This should target the masses to educate them about AMR, its causes, and effects by using more holistic public enlightenment programs [31,37,39–41].

Awareness of the term antibiotic resistance was lower than studies in Kemssie (59.4%) [24], in Nigeria (56.5%) [31], in Romania (85.14%) [42], and in China (95%) [43]. The study done in China was for urban people and those with a higher level of education, but the current study was conducted with respondents having different socio-demographic characteristics. Our study identified about 64 (15.7%) of the respondents were not aware of any term related to AMR. It was almost similar to the national survey in Nigeria (17.0%) [31].

In the present study, about 288 (70.8%) of the respondents were aware that sharing antibiotics with others is a risk factor for AMR. This is significantly higher than a report in Kemssie (17.7%) [24]. Even though better awareness of the risk of sharing antibiotics is observed in the current study, an educational campaign among the public should be given to keep the momentum. The educational campaign should address the reasons for taking antibiotics to only specific individuals prescribed for a particular episode of illness [37]. A higher proportion of the respondents (61.4%) were also aware that failure to complete the course of therapy is a risk for antibiotic resistance, which is higher than the one reported in Kemssie (23.7%) [24]. The educational campaign should also include the importance of taking the full prescription as prescribed [37]. Only about 54.3% of study participants were aware that taking antibiotics without a prescription is a risk factor for AMR. The finding showed an urgent need to revisit rules on antibiotic prescriptions and OTC sales of antibiotics. As there is no strict rule to control the selling of antibiotics without a prescription at the community pharmacy in Ethiopia, antibiotics are used without having been prescribed. This problem could be reduced by educational campaigns for the public and setting up and controlling policies and regulations regarding antibiotic sales [37,39,44]. In addition, involving all pharmacists to educate all patients every time during dispensing antibiotics would be important. Not getting cured from the diseases (77.1%), the need for expensive drugs (63.9%), increased intensity and duration of the diseases (45.9%) and decreased antibiotic activity (45.2%) were reported as the consequences of AMR. The findings were higher than the findings in Kemssie, which were 28.6%, 20.0%, 22.6%, and 23.4%, respectively [24]. The difference might be due to the study setting, where Dessie is highly civilized and a metropolitan City compared to Kemssie.

In the current study, about 238 (58.5%, 95% CI: 53.1–63.1) respondents had good knowledge of AMR. The finding is nearly similar to the study done in Dire Dawa, Ethiopia (62.8%) [45], but higher than other studies conducted in Nigeria (8.3%) [31], and Pakistan (44.4%) [28]. The difference might be attributed to the difference in the questionnaire used, the criteria to say good knowledge and poor knowledge, the study setting and the health administration system employed in each country.

The result showed a lower level of knowledge of AMR among adults, which requires the attention of the stakeholders. Poor educational intervention about AMR among the public, poor focus of health professionals to advise about the risk of AMR, socio-demographic factors and poor attention of health administrators to AMR might be the reasons for lower levels of AMR knowledge [37]. Although overcoming knowledge deficits alone will be insufficient for global AMR behavior change, the authors believe education and awareness campaigns can increase the public’s knowledge of AMR [46,47].

Male gender, educational level, getting advice from health professionals about how to take antibiotics, using health professionals as a source of information on antibiotics, and taking antibiotics without a prescription were significantly associated with good knowledge of AMR.

Males were about 2 times more likely to have good knowledge than females. However, there was no association between the gender of respondents and knowledge of AMR in Nigeria [31]. This difference might be contextual (varied based on setting) and intersect with other socio-demographic factors, particularly education (in Ethiopia, females had lower educational level) and socioeconomic status (females had lower income level) [48]. As the educational level of the majority of females in the country is lower than male, it is likely that AMR knowledge is lower among females. The WHO recommended a need to have a gender and equity focus in all efforts because it is important to national efforts to tackle AMR [49].

In the current study, higher educational levels had a significant association with knowledge of AMR. Study participants with college and above educational levels had 3.5 times better knowledge of AMR and those with grade 11–12 educational levels had 3.7 times better knowledge of AMR than illiterate adults. This is in line with other studies [18,21,26,31,50–55]. The impact of educational training on improving the awareness, knowledge, and perception of AMR is very important [49,54].

The current study showed that participants who reported getting advice from health professionals about how to take antibiotics and using health professionals as sources of information on antibiotics had about 1.8 times and 2.5 times better knowledge than their counterparts, respectively. This is not surprising as health professionals are expected to hold better knowledge about antibiotics and sharing information with individuals could result in better knowledge of the respondents about AMR. Cascading WHO recommendations are crucial to address available information on population diversity to target and refine behavior change among the general population and/or certain occupations [49]. Study participants who ever took antibiotics without a prescription had 1.8 odds of better knowledge of AMR. This might be due to the experience of taking the medication leading to the acquiring of information through different ways like using the internet, medical books and other reading materials that could lead to enhancing knowledge of AMR.

### Limitations of the study

Even though it could report many important findings, the current study had several limitations. Because it was a cross-sectional study, no cause and effect relationships could be established, and the sample size was small because we did not account for design effects. Controlling the difference between the two data collectors was difficult which might result in variations of the data. Even though the tool was used in the previous study in Ethiopia and we established its face validity, the content validity and reliability were not performed to assure tool validity in the current study.

### Conclusion

The current study indicated poor awareness and knowledge of AMR among the adults in Dessie City. Being male, higher educational level, getting advice from health professionals about how to take antibiotics, using health professionals as a source of information on antibiotics, and taking antibiotics without a prescription were significantly associated with good knowledge of AMR. Educational campaigns based on socio-demographic contexts should be provided by the stakeholders to improve the awareness and knowledge of AMR among the public. As health professionals are potential sources of information on AMR, health administrators should plan to use them widely as mediators of information to the public, thereby increasing the awareness and knowledge of AMR among the public.

## Supporting information

S1 Table Socio-demographic characteristics of the study participants among adults in Dessie City, Northeast Ethiopia, 2021 (n = 407). (DOCX)

S2 Table Antibiotic use and related information among the adults of Dessie City, Northeast Ethiopia, 2021 (n = 407). (DOCX)

S3 Table Awareness and knowledge of AMR among the adults of Dessie City, Northeast Ethiopia, 2021 (n = 407). (DOCX)

S4 Table Association factors of knowledge towards AMR among the adults of Dessie City, Northeast Ethiopia, 2021 (n = 407). (DOCX)

S5 Table Annex 1: Questionnaires. (DOCX)

S1 Dataset (SAV)

## Abbreviations

AMR: Antimicrobial Resistance
WHO: World Health Organization
CI: Confidence Interval
