# Supplementary material for: Awareness and knowledge of antimicrobial resistance and factors associated with knowledge among adults in Dessie City, Northeast Ethiopia: Community-based cross-sectional study
Source: PLoS One. 2022 Dec 30;17(12):e0279342. doi: 10.1371/journal.pone.0279342 (PMC9803210; doi:10.1371/journal.pone.0279342)
Supplement: S1 Table — (DOCX) [file pone.0279342.s001.docx]

**S1. Table 1**

| **Variables** | **Categories** | **Frequency n%** |
| --- | --- | --- |
| Gender | Male | 255(62.7) |
|  | Female | 152(37.3) |
| Age category | 20-29 | 98(24.1) |
|  | 30-33 | 95(23.3) |
|  | 34-40 | 116(28.5) |
|  | > 40 | 98(24.1) |
| Residence | Rural | 46(11.3) |
|  | Urban | 361(88.7) |
| Religion | Orthodox | 162(39.8) |
|  | Muslim | 175(43.0) |
|  | Protestant | 52(12.8) |
|  | Catholic | 18(4.4) |
| Marital status | Married | 306(75.2) |
|  | Single | 62(15.2) |
|  | Divorced | 28(6.9) |
|  | Widowed | 11(2.7) |
| Educational level | Unable to read and write | 30(7.4) |
|  | Able to read and write | 82(20.1) |
|  | Grade 8-10 | 69(17.0) |
|  | Grade 11-12 | 123(30.2) |
|  | College and above | 103(25.3) |
| Occupation | Employee | 99(24.3) |
|  | Merchant | 158(38.8) |
|  | Farmer | 13(3.2) |
|  | House wife | 66(16.2) |
|  | Student | 22(5.4) |
|  | No work | 49(12.0) |
| Average monthly income (ETH-Birr) | 0-2500 | 103(25.3) |
|  | 2501-4000 | 108(26.5) |
|  | 4001-6000 | 103(25.3) |
|  | >6000 | 93(22.9) |
