# Supplementary material for: Awareness and knowledge of antimicrobial resistance and factors associated with knowledge among adults in Dessie City, Northeast Ethiopia: Community-based cross-sectional study
Source: PLoS One. 2022 Dec 30;17(12):e0279342. doi: 10.1371/journal.pone.0279342 (PMC9803210; doi:10.1371/journal.pone.0279342)
Supplement: S2 Table — (DOCX) [file pone.0279342.s002.docx]

**S2. Table 2**

| **Variables** | **Categories** | **Frequency n%** |
| --- | --- | --- |
| The last time to take antibiotics | In the last month | 66(16.2) |
|  | In the last 6 months | 115(28.3) |
|  | In the last year | 114(28.0) |
|  | More than a year ago | 94(23.1) |
|  | Cannot remember | 18(4.4) |
| The sources of antibiotics  (More than one answer is possible) | Hospital/healthcare by prescription | 269(66.1) |
|  | Retail outlet pharmacy | 270(66.3) |
|  | From a friend or family member | 117(28.7) |
|  | By sharing with others | 61(15.0) |
| Getting advice from a doctor, nurse or pharmacist on how to take them | Yes | 245(60.2) |
|  | No | 162(39.8) |
| The source of information on antibiotics  (More than one answer is possible) | Healthcare professional | 264(64.9) |
|  | Mass media | 78(19.2) |
|  | Friends/ family | 188(46.2) |
|  | From previous experience | 156(38.3) |
| Ever suffered from different microbial infection during lifetime | Yes | 224(55.0) |
|  | No | 183(45.0) |
| Ever used antibiotics without prescription (self-medication with antibiotics) | Yes | 225(55.3) |
|  | No | 182(44.7) |
| When do you think you should stop taking antibiotics once you’ve begun treatment? (More than one answer is possible) | Don not know | 30(7.4 ) |
|  | When I feel better | 149(36.6) |
|  | When I have taken all the antibiotics as directed. | 291(71.5) |
|  | When I encountered side effects | 108(26.5) |
|  | When forgetting | 21(5.2) |
