# Supplementary material for: Awareness and knowledge of antimicrobial resistance and factors associated with knowledge among adults in Dessie City, Northeast Ethiopia: Community-based cross-sectional study
Source: PLoS One. 2022 Dec 30;17(12):e0279342. doi: 10.1371/journal.pone.0279342 (PMC9803210; doi:10.1371/journal.pone.0279342)
Supplement: S3 Table — (DOCX) [file pone.0279342.s003.docx]

**S3. Table 3**

| Variables | Categories | Frequency | Percent (%) |
| --- | --- | --- | --- |
| When do you think, you should stop taking antibiotics once you have begun treatment? (More than one answer is possible) | Do not know | 30 | 7.4 |
|  | When I feel better | 149 | 36.6 |
|  | When I have taken all the antibiotics as directed. | 291 | 71.5 |
|  | When I encountered side effects | 108 | 26.5 |
|  | When forgetting | 21 | 5.2 |
| Do you heard/encountered the following terms?  (More than one answer is possible) | Antibiotic resistance | 149 | 36.6 |
|  | Drug resistance | 194 | 47.7 |
|  | Antibiotic-resistant bacteria | 237 | 58.2 |
|  | Germs | 299 | 73.5 |
|  | Antimicrobial resistance | 162 | 39.8 |
|  | None of the above | 64 | 15.7 |
| What do you think are risk factors of antimicrobial resistance? (More than one answer is possible) | Over or under use of antibiotic | 178 | 43.7 |
|  | Failure to complete the course of therapy | 250 | 61.4 |
|  | Sharing antibiotics with others | 288 | 70.8 |
|  | Taking antibiotics without prescription | 221 | 54.3 |
|  | Taking antibiotic without considering the dose and time gap | 165 | 40.5 |
|  | I do not know | 60 | 14.7 |
| What do you think are the consequences of the antimicrobial resistance? (More than one answer is possible) | Decrease antibiotic activity | 184 | 45.2 |
|  | Need for expensive drug | 260 | 63.9 |
|  | Not cured from the diseases | 314 | 77.1 |
|  | Increase intensity and duration of the diseases | 187 | 45.9 |
|  | I do not know | 63 | 15.5 |
| knowledge of AMR | Good knowledge | 238 | 58.5 |
|  | Poor knowledge | 169 | 41.5 |
