# Supplementary material for: Awareness and knowledge of antimicrobial resistance and factors associated with knowledge among adults in Dessie City, Northeast Ethiopia: Community-based cross-sectional study
Source: PLoS One. 2022 Dec 30;17(12):e0279342. doi: 10.1371/journal.pone.0279342 (PMC9803210; doi:10.1371/journal.pone.0279342)
Supplement: S4 Table — (DOCX) [file pone.0279342.s004.docx]

**S4. Table 4**

| Variables | Category | Knowledge of AMR | | AOR (95% CI**)** |
| --- | --- | --- | --- | --- |
|  |  | Good % | Poor% |  |
| Gender | Male | 166(65.1) | 89(34.9) | 1.99(1.23,3.20)* |
|  | Female | 72(47.4) | 80(52.6) | 1 |
| Age categories | 20-29 | 63(64.3) | 35(35.7) | 0.95(0.46,1.99) |
|  | 30-33 | 59(62.1) | 36(37.9) | 1.02(0.50,2.05) |
|  | 34-40 | 69(59.5) | 47(40.7) | 1.26(0.66,2.43) |
|  | > 40 | 47(48.0) | 51(52.0) | 1 |
| Educational level | Unable to read and write | 6(20.0) | 24(80.0) | 1 |
|  | Able to read and write | 42(51.2) | 40(48.8) | 2.45(0.83,7.26) |
|  | Grade 8-10 | 36(52.2) | 33(47.8) | 2.52(0.79,8.01) |
|  | Grade 11-12 | 83(67.5) | 40(32.5) | 3.73(1.20,11.61)* |
|  | College and above | 71(68.9) | 32(31.1) | 3.50(1.08,11.39)* |
| Residence | Rural | 21(45.7) | 25(54.3) | 1 |
|  | Urban | 217(60.1) | 144(39.9) | 1.43(0.70,2.92) |
| When did you last take antibiotics | In the last month | 43(65.2) | 23(34.8) | 1.01(0.28,3.61) |
|  | In the last 6 month | 78(67.8) | 37(32.2) | 1.66(0.50,5.51) |
|  | In the last year | 62(54.4) | 52(45.6) | 1.24(0.37,4.11) |
|  | Before a year a go | 48(51.1) | 46(48.9) | 1.04(0.32,3.43) |
|  | Can’t remember | 7(38.9) | 11(61.1) | 1 |
| Getting antibiotics from health facility | Yes | 172(63.9) | 97(36.1) | 1.46(0.89,2.42) |
|  | No | 66(47.8) | 72(52.2) | 1 |
| Getting advice from health professionals about how to take antibiotics | Yes | 171(69.8) | 74(30.2) | 1.84(1.07,3.17)* |
|  | No | 67(41.4) | 95(58.6) | 1 |
| Using health professionals as source of information on antibiotics | Yes | 187(70.8) | 77(29.2) | 2.51(1.48,4.25)* |
|  | No | 51(35.7) | 92(64.3) | 1 |
| Using your previous experience as a source of information on antibiotics | Yes | 103(66.0) | 53(34.0) | 1.10(0.66,1.81) |
|  | No | 135(53.8) | 116(46.2) | 1 |
| Taking antibiotics without prescription | Yes | 158(70.2) | 67(29.8) | 1.86(1.04,3.30)* |
|  | No | 80(44.0) | 102(56.0) |  |
| Suffering from different microbial infections during your life time | Yes | 148(66.1) | 76(33.9) | 1.25(0.70,2.22) |
|  | No | 90(49.2) | 93(50.8) | 1 |
| * *p- Value < 0.05* | | | | |
