# Supplementary material for: Awareness and knowledge of antimicrobial resistance and factors associated with knowledge among adults in Dessie City, Northeast Ethiopia: Community-based cross-sectional study
Source: PLoS One. 2022 Dec 30;17(12):e0279342. doi: 10.1371/journal.pone.0279342 (PMC9803210; doi:10.1371/journal.pone.0279342)
Supplement: S5 Table — (DOCX) [file pone.0279342.s005.docx]

**S5. Annex 1**

**Part 1: Socio-demographic items**

| 101 | Sex | 1. Male 2. Female |
| --- | --- | --- |
| 102 | Age | ____________years |
| 103 | Residence | 1. Rural 2. Urban |
| 104 | Religion | 1. Orthodox 2. Muslim 3. Protestant 4. Catholic 5. Others, please specify _____________ _________ |
| 105 | Marital status | 1. Single 2. Married 3. Divorced 4. Widowed |
| 106 | Education level | 1. Unable to read and write 2. Read and write 3. Grade 8+  4. Grade 12 5. College and above |
| 107 | Occupation | Farmer 2. House hold 3. Merchant 4. Employ 5. Others, please specify _____________ |
| 108 | average monthly income | __________birr |

**Part 2. Items related to antibiotics use**

| 201 | \| Did you take antibiotics without prescription? \| \| --- \| \|  \| | 1. Yes 2. No |
| --- | --- | --- | --- | --- |
|  | Did you Suffer from different microbial infections during your life time? | 1. Yes 2. No |
|  | When did you last take antibiotics? | 1. In the last month  2. In the last 6 months  3. In the last year  4. More than a year ago  5. Never  6. Cannot remember |
| 202 | Where do you get antibiotics? (More than one answer is possible) | 1.hospital/healthcare by prescription  2.Retail outlet pharmacy  3.from a friend or family member  4.By sharing with others |
| 203 | Did you get advice from a doctor, nurse or pharmacist on how to take them? | 1. Yes 2. No 3. Can’t remember |
| 204 | When do you think, you should stop taking antibiotics once you have begun treatment? (More than one answer is possible) | 1. Do not know  2. When I feel better,  3. When I have taken all the antibiotics as directed.  4. When I encountered side effects  5. When forgetting  6. If other, specify ------------------- |

**Part 3: Items to assess awareness about AMR**

| 301 | \| What is your source of information on antibiotics?  (More than one answer is possible) \| \| --- \| \|  \| | 1. Healthcare professional  2. Mass media  3. Friends/ family  4. From previous experience  5. No |
| --- | --- | --- | --- | --- |
| 302 | \| Do you heard/encountered the following terms?  (More than one answer is possible) \| \| --- \| \|  \| | 1. Antibiotic resistance 2. Drug resistance 3. Antibiotic-resistant bacteria 4. Germs 5. Antimicrobial resistance 6. None of the above |
| 303 | What do you think are risk factors of antibiotic resistance? (More than one answer is possible) | 1. Over or under use of antibiotic  2. Failure to complete the course of therapy  3. Sharing antibiotics with others  4. Taking antibiotics without prescription  5. Taking antibiotic without considering the dose and time gap  6.If others, specify___________ _________ |
| 304 | What do you think are the consequences of the antibiotic resistance? (More than one answer is possible) | 1. Decrease antibiotic activity  2. Need for expensive drug  3. Not cured from the diseases  4. Increase intensity and duration of the diseases  5. If others, specify __________________ |

**Part 4: Knowledge of AMR**

| 401 | Antibiotic resistance occurs when your body becomes resistant to antibiotics  and they no longer work as well | 1. True  2. False |
| --- | --- | --- |
| 402 | Many infections are becoming increasingly resistant to treatment by antibiotics | 1. True  2. False |
| 403 | If bacteria are resistant to antibiotics, it can be very difficult or impossible to  treat the infections they cause | 1. True  2. False |
| 404 | Antibiotic resistance is an issue that could affect me or my family | 1. True  2. False |
| 405 | Antibiotic resistance is an issue in other countries but not here | 1. True  2. False |
| 406 | Antibiotic resistance is only a problem for people who take antibiotics regularly | 1. True  2. False |
| 407 | Bacteria which are resistant to antibiotics can be spread from person to person | 1. True  2. False |
| 408 | Antibiotic-resistant infections could make medical procedures like surgery,  organ transplants and cancer treatment much more dangerous | 1. True  2. False |
